# Inhibition of Biofilm Formation by Respiratory Bacterial Pathogens via Silver Nanoparticles and Functionalized HEPA Filters

**DOI:** 10.3390/antibiotics15040370

**Published:** 2026-04-03

**Authors:** Mirella Llamosí, Bruno F. Gomes-Ribeiro, Mónica Echeverry-Rendón, Jose Yuste, Julio Sempere, Mirian Domenech

**Affiliations:** 1 Spanish Pneumococcal Reference Laboratory, National Center for Microbiology, Instituto de Salud Carlos III, 28222 Madrid, Spain; mirella.llamosi@externos.isciii.es (M.L.); jyuste@isciii.es (J.Y.); 2 CIBER de Enfermedades Respiratorias (CIBERES), Instituto de Salud Carlos III, 28029 Madrid, Spain; 3 IMDEA Materials Institute, 28906 Getafe, Spain; bruno.gomes.ribeiro@uj.edu.pl (B.F.G.-R.); monica.echeverry@imdea.org (M.E.-R.)

**Keywords:** HEPA filters, silver nanoparticles, biofilms, *Streptococcus pneumoniae*, *Pseudomonas aeruginosa*, *Staphylococcus aureus*, *Staphylococcus epidermidis*

## Abstract

**Objective**: The objective of this study is to evaluate the ability of silver oxide nanoparticle (Ag_2_ONP)-functionalized high-efficiency particulate air (HEPA) filters and colloidal Ag_2_ONP suspensions to inhibit biofilm formation by major respiratory pathogens causing infections at operating rooms. **Background**: Respiratory infections caused by bacterial pathogens such as *Streptococcus pneumoniae*, *Pseudomonas aeruginosa* and *Staphylococcus* species are often associated with the formation of biofilms, which confer increased resistance to antibiotics and host immune responses. Effective strategies to prevent biofilm formation on biological surfaces and in air filtration systems are urgently needed in clinical settings. **Methods**: The biofilm-forming ability of each bacterial strain was assessed by crystal violet microplate assay, viable count or confocal microscopy after prior incubation of the culture medium with Ag_2_ONP-coated HEPA filter material or colloidal Ag_2_ONP suspension. **Results**: Both silver-functionalized filters and silver nanoparticle suspensions significantly inhibited biofilm formation by *S. pneumoniae* and *P. aeruginosa*, with near-complete suppression observed. In the case of *S. aureus* and *S. epidermidis*, the silver nanoparticle suspension showed partial inhibition of biofilm development. **Conclusions**: Ag_2_ONP-functionalized HEPA filters and colloidal Ag_2_ONP suspensions effectively prevent biofilm formation by major respiratory pathogens, for both Gram-negative and Gram-positive bacteria. These materials show promise for integration with air filtration and surface coating systems to reduce microbial load and transmission in healthcare environments such as operating room facilities.

## 1. Introduction

Airborne transmission of pathogenic microorganisms represents a major public health concern, contributing to diseases such as pneumonia, meningitis, and asthma. Susceptibility varies according to age, occupational exposure, environmental factors, and individual health status [1]. Indoor air filtration via heating, ventilation, and air-conditioning (HVAC) systems is widely used to mitigate exposure to airborne allergens and pathogens [2,3,4]. High-efficiency particulate air (HEPA) filters are deployed in operating rooms and biological laboratories for their superior particle- and microorganism-removal capacity [5]. The protective efficacy of these systems relies on proper installation, regular inspection, and timely filter replacement. Humidity and organic matter accumulation can facilitate microbial adhesion and biofilm formation on ducts and filters, creating persistent reservoirs of pathogens. Poorly maintained HEPA systems may paradoxically contribute to the airborne spread of clinically significant microorganisms and can be a major cause of infections among patients and staff [6,7].

Pathogens such as *Pseudomonas aeruginosa*, *Staphylococcus aureus*, or *Streptococcus pneumoniae* can form biofilms on these filters. As they are damp or poorly ventilated surfaces, the air may be loaded with organic particles such as dust, or there may be an accumulation of debris in these ducts [8,9]. All these aspects enhance the formation of biofilms by these pathogens, increasing the risk of suffering from hospital-associated infections, which are of great concern as these microorganisms growing as biofilms are more resistant to antimicrobial treatment and hospital-grade disinfectant agents used in operating rooms [10].

Beyond treatment, preventive strategies aimed at limiting biofilm establishment are critical, particularly in high-risk clinical environments such as operating rooms and on implantable medical devices, where persistent biofilms can readily develop [11,12]. To mitigate microbial persistence, antimicrobial surface modification strategies have gained considerable attention. Silver and silver oxide nanoparticles (Ag_2_ONPs) exhibit unique physicochemical properties that underpin their broad-spectrum antibacterial activity and position them as promising candidates to address the growing challenge of antimicrobial resistance. Their antimicrobial efficacy is largely associated with the sustained release of Ag^+^ ions, which promote multiple bactericidal mechanisms, including the disruption of membrane integrity, increased permeability, the generation of reactive oxygen species (ROS), the inhibition of protein synthesis, and interference with DNA replication [13]. Accumulating evidence supports the effectiveness of Ag_2_ONPs against a wide range of clinically relevant pathogens, including multidrug-resistant and antibiotic-resistant strains [14,15].

This study aimed to characterize the antibacterial capacity of colloidal Ag_2_ONPs suspensions and HEPA filters functionalized with these Ag_2_ONPs to prevent the growth and biofilm formation of clinically relevant pathogens.

## 2. Results

### 2.1. Antimicrobial Effect of Ag_2_ONP Suspensions Against Biofilms of Different Species

**Figure 1:**
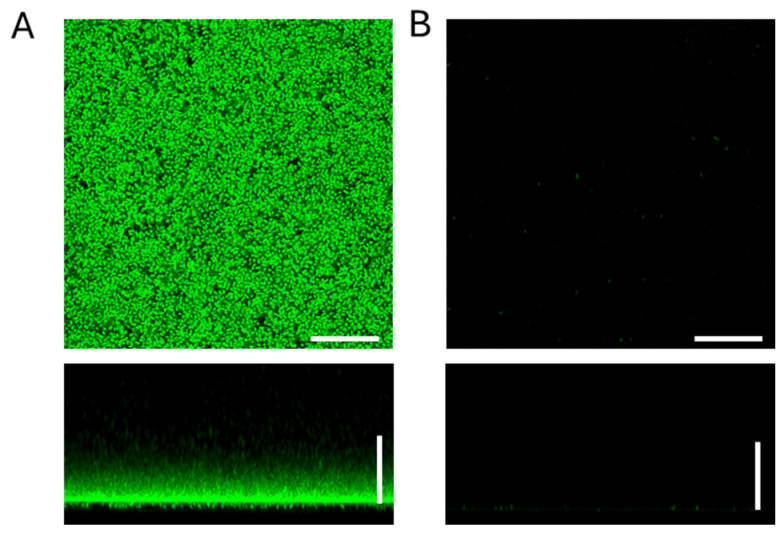
CLSM images of biofilm inhibition of *S. pneumoniae* strain R6 with 0.1 M Ag_2_ONs suspension. The biofilm cells were stained with the *Bac*Light LIVE/DEAD viability kit, with viable cells appearing green (Syto9) and non-viable cells appearing red (IP). (**A**) X–Y projections (images captured at 0.5 μm intervals) and X–Z projections (images captured at 5.0 μm intervals) of strain R6 in C + Y medium (control). (**B**) X–Y projections (images captured at 0.5 μm intervals) and X–Z projections (images captured at 5.0 μm intervals) of strain R6 with 0.1 M AgNP suspension. The bars indicate 25 μm.

The first step was to determine the antibacterial effect of the Ag_2_ONP suspension against the biofilms of four different bacteria. Hence, we tested the 0.1 M Ag_2_ONP suspension evaluating its antimicrobial activity by measuring viable bacterial counts (Table 1) whereas images of biofilm degradation were observed by confocal microscopy (Figure 1). In the case of *S. pneumoniae*, we included two different strains, observing a complete inhibition of biofilm viability compared to the control group lacking Ag_2_ONPs (Table 1).

This strong activity was confirmed by confocal microscopy studies as the bacterial structure of the pneumococcal biofilm was completely destroyed in the presence of the Ag_2_ONPs (Figure 1). The same antimicrobial effect in terms of viable bacterial counts was observed for the *P. aeruginosa* strain with a fully inhibition of the biofilm in the presence of Ag_2_ONPs (Table 1). In the case of methicillin-resistant *S. aureus* (MRSA) and *Staphylococcus epidermidis* strains, we also observed a marked reduction between 3 and 5 Log-units (1.77 × 10^9^ vs. 6.06 × 10^5^ for MRSA and 1.78 × 10^11^ vs. 1.58 × 10^6^ for *S. epidermidis*), although we did not achieve a full inhibition as we obtained for *S. pneumoniae* and *P. aeruginosa* (Table 1).

### 2.2. Ag_2_ONP-Functionalized HEPA Filters Effectively Inhibit Biofilm Formation by Streptococcus pneumoniae and Pseudomonas aeruginosa

**Figure 2:**
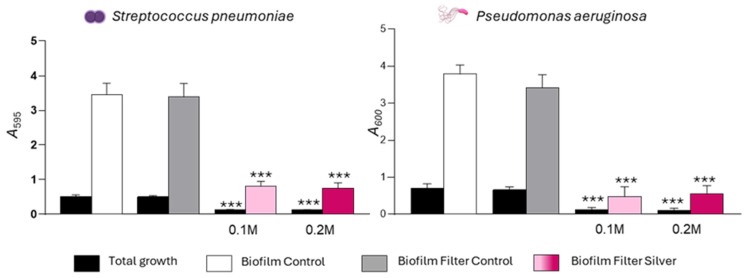
Inhibition of *S. pneumoniae* strain YNM4 (**left**) and *P. aeruginosa* strain ATCC27853 (**right**) biofilm with Ag_2_ONPs-functionalized HEPA filters. C + Y or LB bacterial culture medium was previously incubated at 37 °C under agitation for 24 h with each of the functionalized filters. The strain was inoculated in each of these media, incubated for 5 h at 34 °C or 24 h at 37 °C. The black bars indicate total growth (adherent (biofilm) plus non-adherent (planktonic) cells). The biofilm was processed by staining with crystal violet for 15 min, washed 3 times with distilled water, solubilized with ethanol and then measured for absorbance at 595 or 600 nm. Medium with filter functionalized with 0.2 M silver (Filter Ag 0.2 M; Dark Pink), 0.1 M silver (Filter Ag 0.1 M; Light Pink), unfunctionalized filter (Filter Control; Grey) and medium without filter (Control; White). Assays were performed in triplicates, including at least three independent experiments. Standard deviation bars are shown, and asterisks mark results that are statistically significant (two-tailed Student’s *t* test: ***, *p* < 0.001).

The antimicrobial activity of Ag_2_ONP-functionalized HEPA filters (Figure 2) was evaluated using *S. pneumoniae* and *P. aeruginosa* selected as representative Gram-positive and Gram-negative respiratory pathogens associated with biofilm formation. Two silver concentrations (0.1 and 0.2 M) were tested. Silver functionalization markedly affected bacterial growth. Complete growth inhibition was observed for both pathogens, even at the lower concentration (0.1 M) (Figure 2).

The impact of HEPA filters on biofilm formation was also assessed. Control filters lacking silver functionalization showed no effect on bacterial growth or biofilm development. In contrast, Ag_2_ONP-functionalized HEPA filters resulted marked reduced biofilm formation for both *S. pneumoniae* and *P. aeruginosa* (Figure 2).

## 3. Discussion

**Figure 3:**
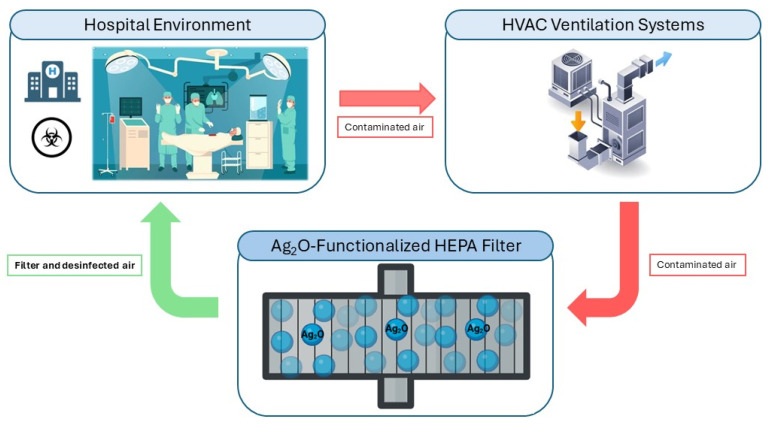
Diagram showing how silver oxide nanoparticle-based filters in HVAC can help improve air quality in the hospital environment. Created by BioRender.com.

The treatment of bacterial biofilms remains a major clinical challenge due to their intrinsic tolerance to antimicrobial agents and their enhanced capacity to evade host immune responses compared with planktonic cells [16]. Conventional antibiotics, when used alone, are often ineffective against established biofilms and may contribute to therapeutic failure. Consequently, alternative antibiofilm strategies have been explored, including enzibiotics, antioxidant or nanoparticles–antibiotic combinations, and nanoparticle-based approaches [17,18]. For example, AgNPs combined with antibiotics have demonstrated efficacy against bacterial biofilms in chronic rhinosinusitis models [19] and to inhibit the growth of the different bacterial pathogens [20,21]. Previous studies suggest that combining physical filtration with active biocidal properties represents a promising strategy to minimize biofilm formation on inert surfaces. For example, chlorhexidine-coated air filters and nanocellulose filters incorporating silver and MXene particles have demonstrated broad-spectrum antimicrobial activity against bacteria, fungi, and viruses [22,23]. Similarly, AgNP biocomposites embedded in microfibrillated cellulose have shown bactericidal activity against both susceptible and resistant strains, offering environmentally adaptable options for surface coatings and medical devices [24]. In this context, HEPA filters functionalized with metal oxide agents have been reported to inhibit the growth of *S. pneumoniae* and *P. aeruginosa*, highlighting their potential role in reducing microbial burden in controlled environments [25]. Therefore, the implementation of silver oxide nanoparticle-based filters in HVAC systems to improve air quality in operating rooms and hospital environments is feasible (Figure 3), as this system would be capable to prevent biofilm formation by clinically relevant pathogens.

The present study demonstrates that Ag_2_ONP suspensions and Ag_2_ONP-functionalized HEPA filters effectively prevent biofilm formation, particularly in *S. pneumoniae* and *P. aeruginosa*. The Ag_2_ONP suspension achieved complete inhibition of biofilm development in both species, whereas functionalized filters produced a marked but comparatively lower inhibitory effect. This difference may be attributed to the higher immediate bioavailability of Ag^+^ ions in suspension, facilitating direct and sustained interaction with bacterial cells. In contrast, silver immobilized on filter surfaces likely provides a slower or more localized ion release. While this controlled release may be advantageous in airflow systems such as hospital operating rooms, it may exhibit reduced activity against mature or structurally complex biofilms [26].

The Ag_2_ONP suspension also inhibited biofilm formation by MRSA and *S. epidermidis*, although to a lesser extent than observed for *S. pneumoniae* and *P. aeruginosa*. These differences may be explained by variations in biofilm matrix composition and architecture. Biofilms formed by *Staphylococcus* spp. are characterized by dense intercellular polysaccharide networks with limited pore size, which may restrict nanoparticle diffusion [27,28]. In contrast, the water-channel architecture of *P. aeruginosa* biofilms [29] and the comparatively hydrated, eDNA- and protein-rich matrix of *S. pneumoniae* facilitates greater nanoparticle penetration [30].

A key limitation of this study is the use of a static in vitro biofilm model that evaluates the activity of medium conditioned by contact with the filters, rather than their performance under dynamic airflow conditions, thereby highlighting a gap between the in vitro model and real-world air filtration applications. Although this approach enables controlled assessment of antimicrobial activity, it does not fully replicate dynamic clinical conditions, such as continuous airflow, fluctuations in humidity, or interactions with host tissues and polymicrobial communities [31,32,33]. Future studies should focus on optimizing filter functionalization to achieve controlled and sustained silver ion release, mechanical stress, or disposal processes could potentially lead to particle or ion release. Therefore, while our results demonstrate promising antibacterial and antibiofilm performance, further studies are needed to evaluate long-term stability, potential Ag^+^ release, and environmental impact under realistic operating conditions [34]. Validation under dynamic conditions, such as airflow chambers or simulated hospital environments, will be essential to determine real-world applicability. Additionally, integrating synergistic agents—including antimicrobial peptides, biofilm-dispersing enzymes, or nanoparticles containing complementary metals—may enhance antibiofilm efficacy while reducing the silver concentration required, thereby minimizing potential toxicological concerns and production costs [35].

Overall, our findings support the potential of Ag_2_ONP-functionalized HEPA filters as a preventive strategy to reduce bacterial colonization and biofilm formation in settings where air quality and surface sterility are critical, including hospitals, operating rooms, and medical ventilation systems. The strong antibiofilm activity observed with Ag_2_ONP suspensions, even against resistant strains such as MRSA, further highlights their potential as complementary antimicrobial agents, although their application beyond air filtration systems requires further investigation.

## 4. Materials and Methods

### 4.1. Strains, Media and Culture Conditions

For this study we used five strains of Gram-positive bacteria and one Gram-negative bacterial strain (Table 2). Among the five Gram-positive strains, three of them were *S. pneumoniae*, and the other two were *S. aureus* and *S. epidermidis*.

For broth cultures, the pneumococcal strains were grown in CpH8 medium supplemented with 0.08% yeast extract (C + Y medium), Staphylococcal strains were grown in Tryptic Soy Broth medium supplemented with glucose (0.4%) and yeast extract (0.3%) (TSBGY medium) and the *P. aeruginosa* strain was grown in Luria–Bertani broth (LB medium). Growth was controlled by measuring the optical density at 550 nm for *S. pneumoniae*, *S. aureus* and *S. epidermidis* strains, whereas for *P. aeruginosa* we measured at 600 nm. All strains were preserved in their specific medium and stored in frozen aliquots at −80 °C.

### 4.2. Filter Functionalization, Characterization and Preparation of AgNP Suspensions

Commercially available H13 (EN 1822) HEPA filter (Venfilter, Barcelona, Spain) and 0.1 M and 0.2 M colloidal suspensions of Ag_2_ particles, were prepared, functionalized, and characterized as previously described [25]. Once the particles were collected through vacuum filtration, they were washed twice with dH_2_O and then dried at 150 °C for 1 h in vacuum to evaporate any residual water and convert any resulting hydroxide into the oxide form. Ag_2_ONPs were produced by chemical reduction of silver nitrate (AgNO_3_) with potassium hydroxide (KOH), and 0.1 M and 0.2 M colloidal suspensions in DI H_2_O were prepared to a final volume of 25 mL. Glass Fibre HEPA filter media was cut and removed in a clean environment, cleaned with a nitrogen (N_2_) stream and positioned over filter paper with tape. The spraying process was carried out with a mini-handheld spray gun (Sagola 474, Vitoria-Gasteiz, Spain), at a normal position with respect to the filter media, distanced at approximately 15 cm height from surface. Each colloidal suspension was sprayed onto the filter for 10 s per area, with two repetitions. After coating deposition, the filter dried in air at room temperature and then was cut into 2 × 2 cm^2^ squares for further characterization. Sample morphology was characterized by scanning electron microscopy (Apreo 2, ThermoFischer Scientific, Waltham, MA, USA) at low vacuum and an applied voltage of 5–10 kV. Chemical composition was determined by energy dispersive spectroscopy (EDS, UltimMax 40 from Oxford Instruments (Oxford, UK), using AZtec Software for acquisition and data processing).

Prior to biological testing, all samples were sterilized using low-temperature (<40 °C) ethylene oxide in an ANPROLENE AN75 sterilizer (Andersen Sterilizers, Haw River, NC, USA), with a gas dwell time of approximately 12 h, relative humidity of ~50%, using EO cartridges containing 87% ethylene oxide as the active ingredient.

### 4.3. Biofilm Inhibition Tests

Filter extracts were prepared by incubating the different filters (functionalized with Ag_2_ONPs at 0.1 M and 0.2 M, as well as non-functionalized control filters) in culture media (LB, TSBGY, or C + Y) for 24 h at 37 °C under shaking conditions. After incubation, the resulting media, containing soluble components released from the filters, were collected and used as filter extracts for subsequent experiments. The strains were incubated at 37 °C in the corresponding medium (LB, TSBGY or C + Y) to an optical density of 0.5–0.6 at 550 nm for *S. pneumoniae*, *S. aureus*, and *S. epidermidis* strains and to an optical density of 1 at 600 nm for *P. aeruginosa* strain. The culture was then diluted to an optical density corresponding to 4.5 × 10^6^ Colony-Forming Unit (CFU)/mL in fresh medium with filter extract (previously incubated with both the functionalized filters or non-functionalized filter) and in fresh medium without filters as control. Then, 200 μL per well was added to a 96-well polystyrene microtiter plate (Costar 3595; Corning, Corning, NY, USA), and plates were incubated 5 h at 34 °C for pneumococcal strains and 24 h at 37 °C for all other strains. After the incubation, the biofilm was processed by staining with crystal violet as previously described [36,37]. Briefly, total growth (adhered and not adhered cells) was measured (595 nm by *S. pneumoniae*/600 nm by *P. aeruginosa*) using the BioTek Epoch 2 reader (Winooski, VT, USA) and then CV (1%) was added for 15 min, washed three times with distilled water, solubilized with ethanol and then measured the biofilm biomass at 595 nm (*A*_595_) or 600 nm (*A*_600_).

In addition, the Ag_2_ONP suspensions with which the filters were functionalized were used to perform biofilm inhibition tests in which cell viability was determined. For this purpose, the 0.2 M Ag_2_ONP suspension was incubated with the corresponding culture medium as previously described in volume–volume ratio at 37 °C for 24 h with shaking. Thus, the silver concentration would be 0.1 M. A non-preincubated culture medium was used as a control. The supernatant of this suspension or non-preincubated culture medium was then used for the biofilm inhibition test. After the corresponding incubations of the biofilm, the supernatant was removed, 100 µL per well of PBS was added, the biofilm was homogenized, and serial dilutions were made and subsequently plated on blood agar, Tryptic Soy Agar, or LB plates.

### 4.4. Confocal Laser Scanning Microscopy

For CLSM assays, *S. pneumoniae* strain R6 was used. A culture of the strain grown in its corresponding medium to an optical density of 0.5–0.6 at 550 nm and diluted on one side in the same medium as the control and on the other side in the medium pre-incubated with the silver oxide nanoparticles to have 4.5 × 10^6^ CFU/mL was inoculated in 200 µL on a plate (µ-Slide 4 Well Glass Bottom, Ibidi, Gräfelfing, Germany). They were incubated at 37 °C for 24 h. After the incubation period, the sample was washed with water and stained for 15 min at room temperature with the *Bac*Light LIVE/DEAD viability kit, with viable cells appearing green (Syto9) and non-viable cells appearing red (IP). We observed the samples with the Leica spectral SP5 confocal microscope at 1024 × 1024 700 Hz; the images were analyzed using the LAS AF software.

### 4.5. Statistical Analysis

Data presented represent results obtained from at least three repeated independent experiments, representing at least three replicates. Statistical analyses were performed using GraphPad InStat v. 8.0 (GraphPad Software, La Jolla, CA, USA). For comparison of two groups, we used the two-tailed Student’s *t*-test. Differences were considered statistically significant at *, *p* < 0.001.

## Figures and Tables

**Table 1:** Inhibition of biofilm of different strains with 0.1 M Ag_2_ONP suspension.

Bacteria	Strain	Viability Control (CFU/mL)	Viability 0.1 M Ag_2_ONPs (CFU/mL)	*p*-Value ^1^
*Streptococcus pneumoniae*	YNM4	6.51 × 10^8^	<LOD	***
	ATCC49619	1.11 × 10^9^	<LOD	***
*Pseudomonas aeruginosa*	ATCC27853	2.16 × 10^9^	<LOD	***
*Staphylococcus aureus*	MRSA 60335/19	1.77 × 10^9^	6.06 × 10^5^	***
*Staphylococcus epidermidis*	CECT4184	1.78 × 10^11^	1.58 × 10^6^	***

^1^ Comparison between the control and the 0.1 M Ag_2_ONPs treatment. Asterisk mark results that are statistically significant (two-tailed Student’s *t* test: ***, *p* < 0.001). Note: CFU, colony forming units; <LOD, below the limit of detection (1 × 10^2^ CFU/mL).

**Table 2:** Strains used in this study with their description.

Bacteria	Strain	Description *
*Streptococcus pneumoniae*	YNM4	Transformant M11 with DNA from strain 1228/19; serotype 19A; Laboratory strain
*Streptococcus pneumoniae*	ATCC49619	Sputum; serotype 19F; ATCC
*Streptococcus pneumoniae*	R6	Non-capsulated strain; Laboratory strain
*Pseudomonas aeruginosa*	ATCC27853	Blood; ATCC
*Staphylococcus aureus*	MRSA 60335/19	Blood; serotype 8; [36]
*Staphylococcus epidermidis*	CECT4184	Catheter sepsis; CECT

* Serotype; Source/Reference. Note: CECT (Spanish Collection of Standard Crops, Valencia, Spain); ATCC (American Type Culture Collection, Manassas, VA, USA).

## Data Availability

All epidemiological and experimental data requests should be submitted to J.S. (jsempere@isciii.es) or M.D. (miriam.domenech@isciii.es). Requests will be assessed for scientific rigour before being granted, and a data-sharing agreement might be required.

## Abbreviations

The following abbreviations are used in this manuscript:

AgNPs	Silver Nanoparticles
ATCC	American Type Culture Collection
CECT	Spanish Collection of Standard Crops
CFU	Colony-Forming Unit
CLSM	Confocal laser scanning microscopy
C + Y	CpH8 medium supplemented with 0.08% yeast extract
HEPA	High-Efficiency Particulate Air
LB	Luria–Bertani broth
MRSA	Methicillin-resistant *Staphylococcus aureus*
TSBGY	Tryptic Soy Broth medium supplemented with glucose (0.4%) and yeast extract (0.3%)

## References

[1] Al-Shaarani A.A.Q.A., Pecoraro L. (2024). A review of pathogenic airborne fungi and bacteria: Unveiling occurrence, sources, and profound human health implication. Front. Microbiol..

[2] Bolashikov Z.D., Melikov A.K. (2009). Methods for air cleaning and protection of building occupants from airborne pathogens. Build. Environ..

[3] Gherasim A., de Blay F. (2020). Does Air Filtration Work for Cat Allergen Exposure?. Curr. Allergy Asthma Rep..

[4] Vijayan V., Paramesh H., Salvi S., Dalal A.K. (2015). Enhancing indoor air quality—The air filter advantage. Lung India.

[5] Mittal H., Parks S.R., Pottage T., Walker J.T., Bennett A.M. (2011). Survival of Microorganisms on HEPA Filters. Appl. Biosaf..

[6] Lee S.-T., Liang C.-C., Chien T.-Y., Wu F.-J., Fan K.-C., Wan G.-H. (2018). Effect of ventilation rate on air cleanliness and energy consumption in operation rooms at rest. Environ. Monit. Assess..

[7] Wu H.T., Li Q.S., Dai R.C., Liu S., Wu L., Mao W., Ji C.H. (2021). Effects of air-conditioning systems in the public areas of hospitals: A scoping review. Epidemiol. Infect..

[8] Patra S., Saha S., Singh R., Tomar N., Gulati P. (2025). Biofilm battleground: Unveiling the hidden challenges, current approaches and future perspectives in combating biofilm associated bacterial infections. Microb. Pathog..

[9] Ortiz G., Yagüe G., Segovia M., Catalán V. (2009). A Study of Air Microbe Levels in Different Areas of a Hospital. Curr. Microbiol..

[10] Abdelhamid A.G., Yousef A.E. (2023). Combating Bacterial Biofilms: Current and Emerging Antibiofilm Strategies for Treating Persistent Infections. Antibiotics.

[11] Falde E.J., Yohe S.T., Colson Y.L., Grinstaff M.W. (2016). Superhydrophobic materials for biomedical applications. Biomaterials.

[12] Paul S., Rao L., Stein L.H., Salemi A., Mitra S. (2023). Development of a Carbon Nanotube-Enhanced FAS Bilayer Amphiphobic Coating for Biological Fluids. Nanomaterials.

[13] Karataş H., Eker F., Akdaşçi E., Bechelany M., Karav S. (2026). Silver Nanoparticles in Antibacterial Research: Mechanisms, Applications, and Emerging Perspectives. Int. J. Mol. Sci..

[14] Xie N. (2024). Synthesis and antibacterial effects of silver nanoparticles (AgNPs) against multi-drug resistant bacteria. Biomed. Mater. Eng..

[15] de Lacerda Coriolano D., de Souza J.B., Bueno E.V., dos Santos Medeiros S.M.d.F.R., Cavalcanti I.D.L., Cavalcanti I.M.F. (2021). Antibacterial and antibiofilm potential of silver nanoparticles against antibiotic-sensitive and multidrug-resistant Pseudomonas aeruginosa strains. Braz. J. Microbiol..

[16] Domenech M., Ramos-Sevillano E., García E., Moscoso M., Yuste J. (2013). Biofilm formation avoids complement immunity and phagocytosis of *Streptococcus pneumoniae*. Infect. Immun..

[17] Domenech M., García E., Moscoso M. (2011). In Vitro Destruction of *Streptococcus pneumoniae* Biofilms with Bacterial and Phage Peptidoglycan Hydrolases. Antimicrob. Agents Chemother..

[18] Vázquez R., García P. (2019). Synergy Between Two Chimeric Lysins to Kill *Streptococcus pneumoniae*. Front. Microbiol..

[19] Feizi S., Cooksley C.M., Nepal R., Psaltis A.J., Wormald P.-J., Vreugde S. (2022). Silver nanoparticles as a bioadjuvant of antibiotics against biofilm-mediated infections with methicillin-resistant *Staphylococcus aureus* and *Pseudomonas aeruginosa* in chronic rhinosinusitis patients. Pathology.

[20] Khatoon N., Alam H., Khan A., Raza K., Sardar M. (2019). Ampicillin Silver Nanoformulations against Multidrug resistant bacteria. Sci. Rep..

[21] Halawani E.M., Hassan A.M., Gad El-Rab S.M. (2020). Nanoformulation of Biogenic Cefotaxime-Conjugated-Silver Nanoparticles for Enhanced Antibacterial Efficacy Against Multidrug-Resistant Bacteria and Anticancer Studies. Int. J. Nanomed..

[22] Qin X., Xiong Y., Xuan S., Kang J., Wang D., Wang H., Wang L., Wu Z. (2024). Nanocellulose-reinforced air filter with gradient hierarchical structure for highly effective and reuseable antibacterial air filtration. J. Membr. Sci..

[23] Watson R., Oldfield M., Bryant J.A., Riordan L., Hill H.J., Watts J.A., Alexander M.R., Cox M.J., Stamataki Z., Scurr D.J. (2022). Efficacy of antimicrobial and anti-viral coated air filters to prevent the spread of airborne pathogens. Sci. Rep..

[24] Garza-Cervantes J.A., Mendiola-Garza G., de Melo E.M., Dugmore T.I.J., Matharu A.S., Morones-Ramirez J.R. (2020). Antimicrobial activity of a silver-microfibrillated cellulose biocomposite against susceptible and resistant bacteria. Sci. Rep..

[25] Ribeiro B., Vázquez-López A., Vazquez-Pufleau M., Llamosí M., Sempere J., Yuste J., Domenech M., Wang D.Y., Vilatela J.J., Llorca J. (2024). Control of microbial agents by functionalization of commercial air filters with metal oxide particles. Mater. Chem. Phys..

[26] Gallus I.J., Boyraz E., Maryška J. (2023). Antimicrobial Properties of Nanofiber Membrane and Commercial Micromembrane by Modification with Diethylenetriamine (DETA) and Attachment of Silver Nanoparticles. J. Nanomater..

[27] Stewart P.S. (2003). Diffusion in Biofilms. J. Bacteriol..

[28] Feng Q.L., Wu J., Chen G.Q., Cui F.Z., Kim T.N., Kim J.O. (2000). A mechanistic study of the antibacterial effect of silver ions on *Escherichia coli* and *Staphylococcus aureus*. J. Biomed. Mater. Res..

[29] Karavolos M.H., Winzer K., Williams P., Khan C.M.A. (2013). Pathogen espionage: Multiple bacterial adrenergic sensors eavesdrop on host communication systems. Mol. Microbiol..

[30] Bibbs R.K., Harris R.D., Peoples V.A., Barnett C., Singh S.R., Dennis V.A., Coats M.T. (2014). Silver polyvinyl pyrrolidone nanoparticles exhibit a capsular polysaccharide influenced bactericidal effect against *Streptococcus pneumoniae*. Front. Microbiol..

[31] Bjarnsholt T., Alhede M., Alhede M., Eickhardt-Sørensen S.R., Moser C., Kühl M., Jensen P.Ø., Høiby N. (2013). The in vivo biofilm. Trends Microbiol..

[32] Malysheva A., Ivask A., Doolette C.L., Voelcker N.H., Lombi E. (2021). Cellular binding, uptake and biotransformation of silver nanoparticles in human T lymphocytes. Nat. Nanotechnol..

[33] Gao X., Li R., Yourick J.J., Sprando R.L. (2022). Transcriptomic and proteomic responses of silver nanoparticles in hepatocyte-like cells derived from human induced pluripotent stem cells. Toxicol. In Vitro.

[34] Li F., Tan J., Yang Q., He M., Yu R., Liu C., Zhou X. (2022). Multi-Endpoint Toxicity Tests and Effect-Targeting Risk Assessment of Surface Water and Pollution Sources in a Typical Rural Area in the Yellow River Basin, China. Chemosensors.

[35] Naskar A., Kim K. (2019). Nanomaterials as Delivery Vehicles and Components of New Strategies to Combat Bacterial Infections: Advantages and Limitations. Microorganisms.

[36] Sempere J., Llamosí M., Román F., Lago D., González-Camacho F., Pérez-García C., Yuste J. (2022). Clearance of mixed biofilms of *Streptococcus pneumoniae* and methicillin-susceptible/resistant *Staphylococcus aureus* by antioxidants N-acetyl-l-cysteine and cysteamine. Sci. Rep..

[37] Llamosí M., Sempere J., Coronel P., Gimeno M., Yuste J., Domenech M. (2022). Combination of Cefditoren and N-acetyl-l-Cysteine Shows a Synergistic Effect against Multidrug-Resistant *Streptococcus pneumoniae* Biofilms. Microbiol. Spectr..

